# Supplementary material for: The clinical impact of comorbidities among patients with idiopathic pulmonary fibrosis undergoing anti-fibrotic treatment: A multicenter retrospective observational study
Source: PLoS One. 2023 Sep 19;18(9):e0291489. doi: 10.1371/journal.pone.0291489 (PMC10508598; doi:10.1371/journal.pone.0291489)
Supplement: S1 Fig — (PPTX) [file pone.0291489.s002.pptx]

## Slide 1
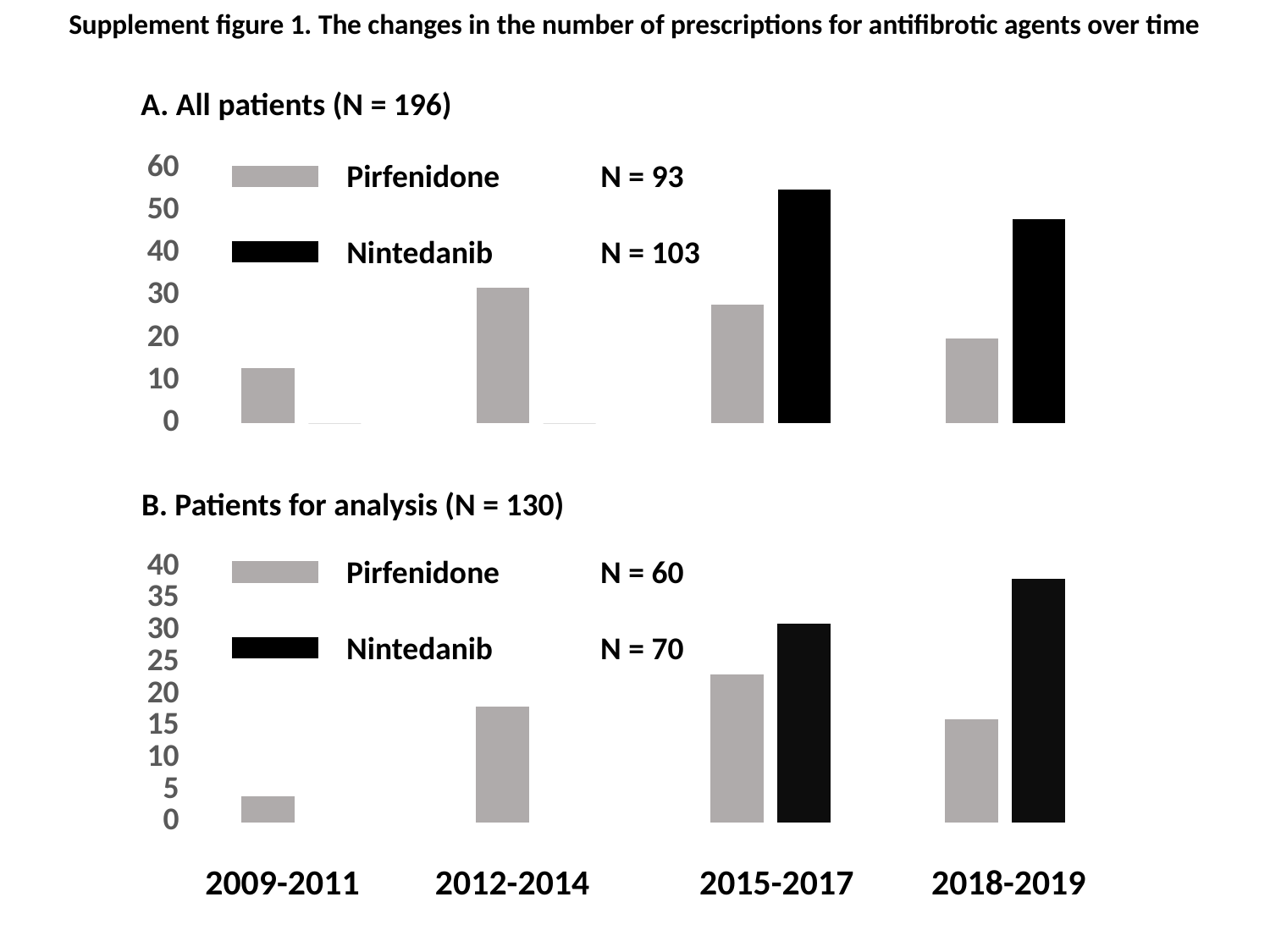

Supplement figure 1. The changes in the number of prescriptions for antifibrotic agents over time
A. All patients (N = 196)
### Chart
| Category | P | N |
|---|---|---|
| 2009-2011 | 13.0 | 0.0 |
| 2012-2014 | 32.0 | 0.0 |
| 2015-2017 | 28.0 | 55.0 |
| 2018-2019 | 20.0 | 48.0 |	Pirfenidone	N = 93
	Nintedanib	N = 103
B. Patients for analysis (N = 130)
### Chart
| Category | P | N |
|---|---|---|
| ① | 4.0 | None |
| ② | 18.0 | None |
| ③ | 23.0 | 31.0 |
| ④ | 16.0 | 38.0 |	Pirfenidone	N = 60
	Nintedanib	N = 70
2009-2011
2012-2014
2015-2017
2018-2019
