# Supplementary material for: The clinical impact of comorbidities among patients with idiopathic pulmonary fibrosis undergoing anti-fibrotic treatment: A multicenter retrospective observational study
Source: PLoS One. 2023 Sep 19;18(9):e0291489. doi: 10.1371/journal.pone.0291489 (PMC10508598; doi:10.1371/journal.pone.0291489)
Supplement: S2 Fig — Comparison of the curves by log-rank test in 196 patients according to CCIS group (A), and according to CCIS group in patients treated with nintedanib (B) and pirfenidone (C) showed no significant difference (P = 0.75, P = 0.43, and P = 0.62, respectively). Abbreviations: CCIS, Charlson Comorbidity Index Score; IPF, idiopathic pulmonary fibrosis. (PPTX) [file pone.0291489.s003.pptx]

## Slide 1
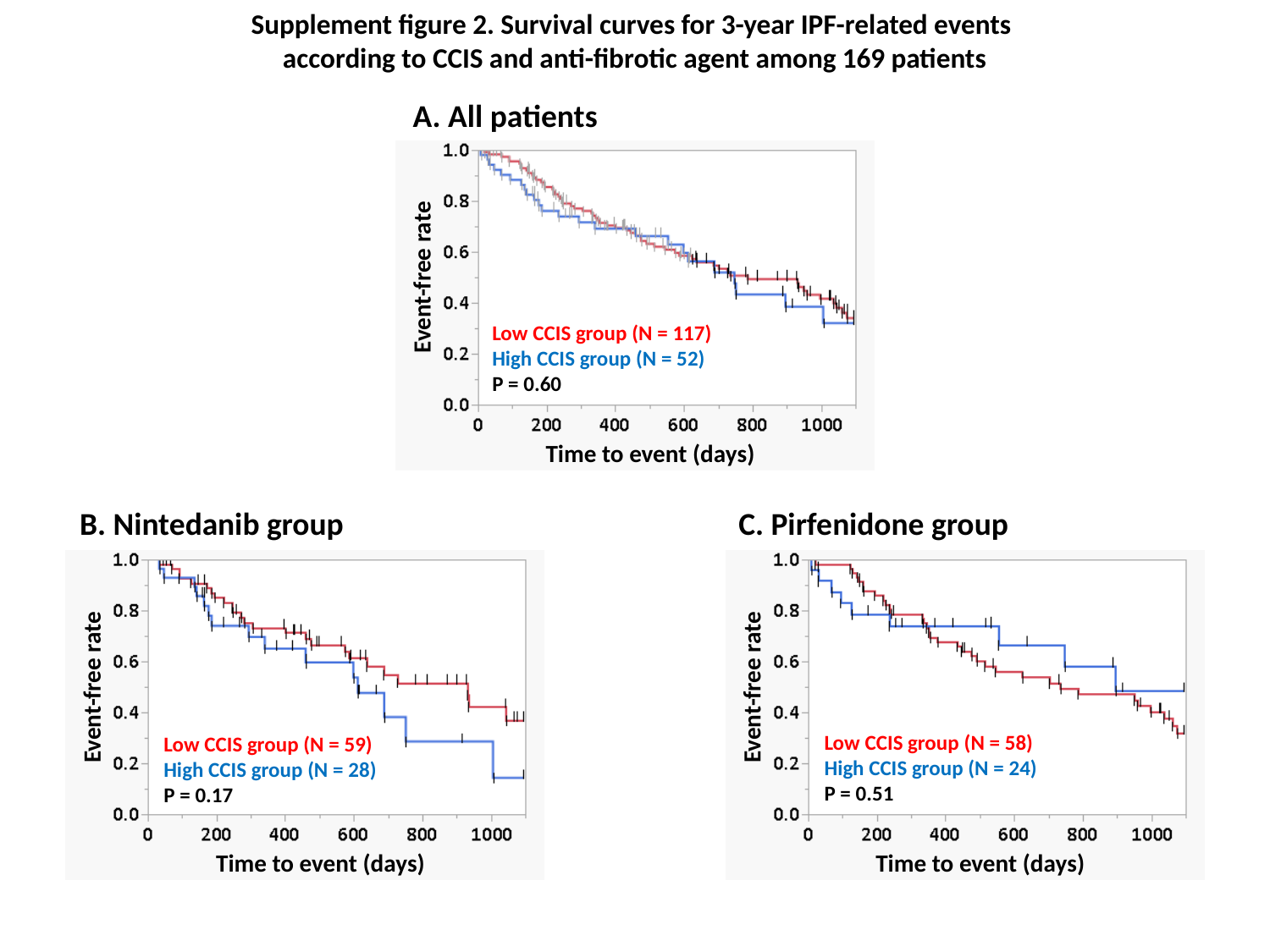

Supplement figure 2. Survival curves for 3-year IPF-related events
according to CCIS and anti-fibrotic agent among 169 patients
A. All patients
Event-free rate
Low CCIS group (N = 117)
High CCIS group (N = 52)
P = 0.60
Time to event (days)
B. Nintedanib group
C. Pirfenidone group
Event-free rate
Event-free rate
Low CCIS group (N = 58)
High CCIS group (N = 24)
P = 0.51
Low CCIS group (N = 59)
High CCIS group (N = 28)
P = 0.17
Time to event (days)
Time to event (days)
