# Supplementary material for: The clinical impact of comorbidities among patients with idiopathic pulmonary fibrosis undergoing anti-fibrotic treatment: A multicenter retrospective observational study
Source: PLoS One. 2023 Sep 19;18(9):e0291489. doi: 10.1371/journal.pone.0291489 (PMC10508598; doi:10.1371/journal.pone.0291489)
Supplement: S3 Fig — Comparison of the curves by log-rank test in 130 patients according to CCIS group (A), and according to CCIS group in patients treated with nintedanib (B) and pirfenidone (C) showed no significant difference (P = 0.30, P = 0.21, and P = 0.91, respectively). Abbreviations: CCIS, Charlson Comorbidity Index Score; IPF, idiopathic pulmonary fibrosis. (PPTX) [file pone.0291489.s004.pptx]

## Slide 1
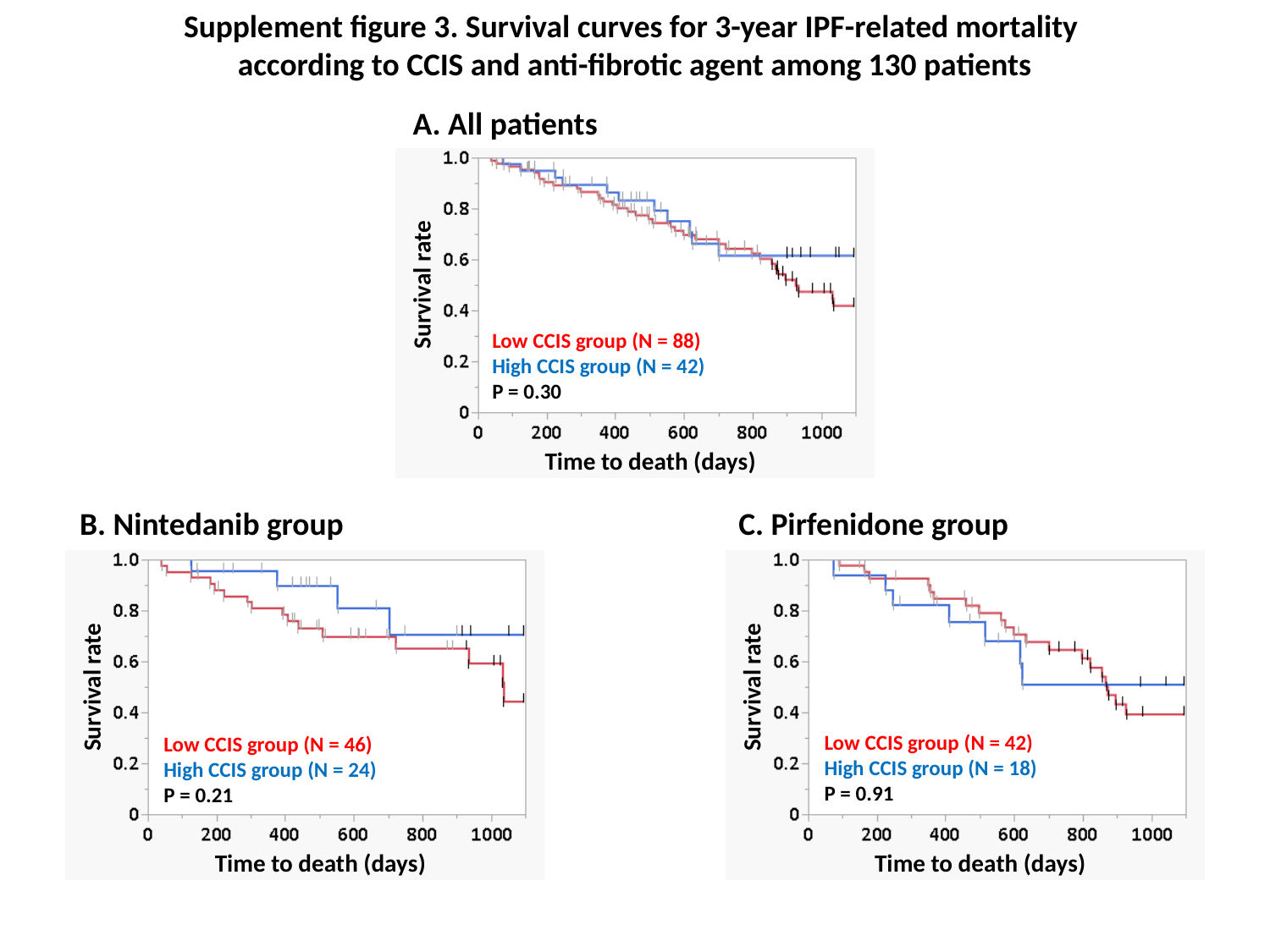

Supplement figure 3. Survival curves for 3-year IPF-related mortality
according to CCIS and anti-fibrotic agent among 130 patients
A. All patients
Survival rate
Low CCIS group (N = 88)
High CCIS group (N = 42)
P = 0.30
Time to death (days)
B. Nintedanib group
C. Pirfenidone group
Survival rate
Survival rate
Low CCIS group (N = 42)
High CCIS group (N = 18)
P = 0.91
Low CCIS group (N = 46)
High CCIS group (N = 24)
P = 0.21
Time to death (days)
Time to death (days)
