# Supplementary material for: The clinical impact of comorbidities among patients with idiopathic pulmonary fibrosis undergoing anti-fibrotic treatment: A multicenter retrospective observational study
Source: PLoS One. 2023 Sep 19;18(9):e0291489. doi: 10.1371/journal.pone.0291489 (PMC10508598; doi:10.1371/journal.pone.0291489)
Supplement: S1 Table — (PPTX) [file pone.0291489.s005.pptx]

## Slide 1
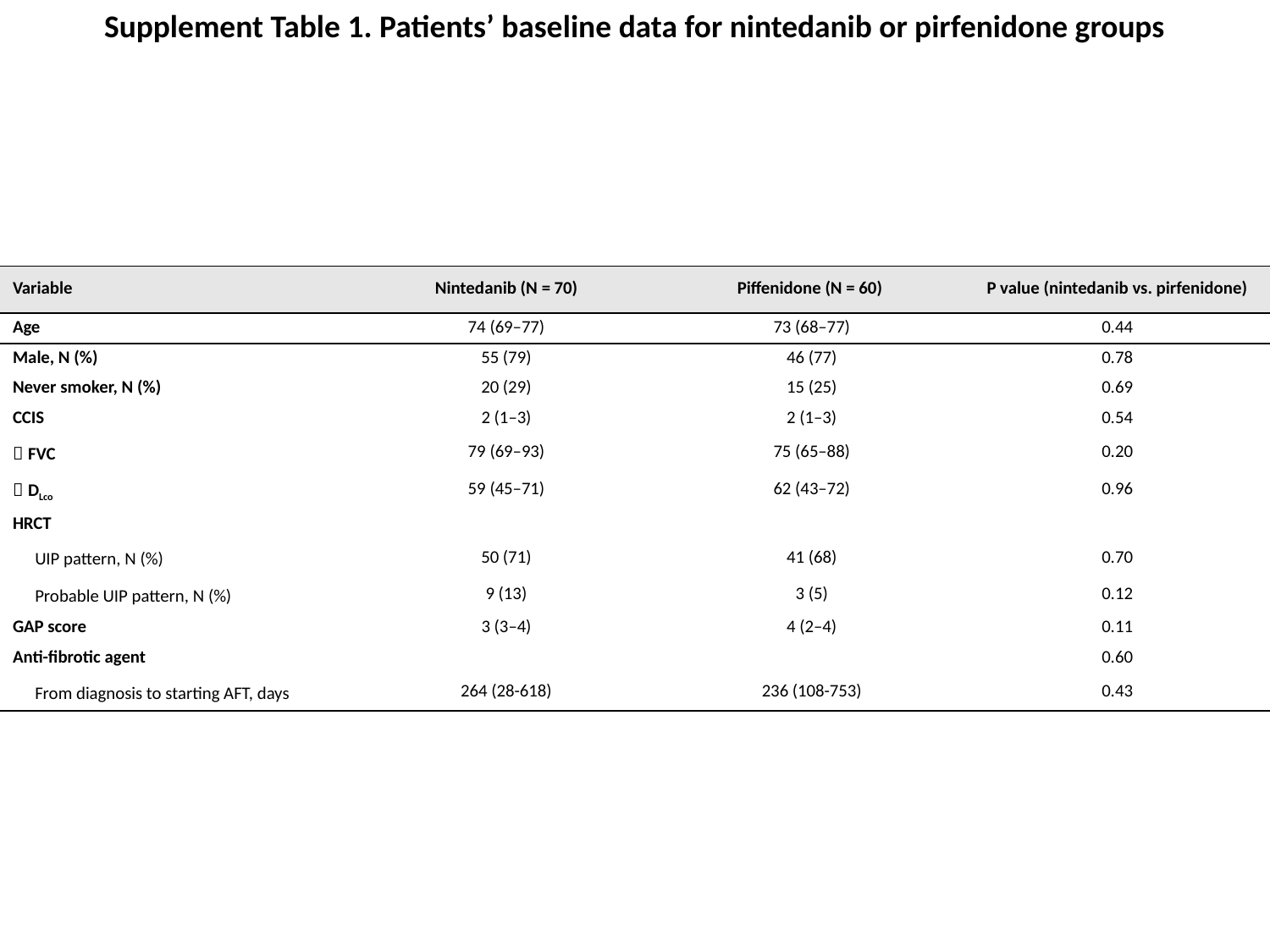

Supplement Table 1. Patients’ baseline data for nintedanib or pirfenidone groups
| Variable | Nintedanib (N = 70) | Piffenidone (N = 60) | P value (nintedanib vs. pirfenidone) |
| --- | --- | --- | --- |
| Age | 74 (69–77) | 73 (68–77) | 0.44 |
| Male, N (%) | 55 (79) | 46 (77) | 0.78 |
| Never smoker, N (%) | 20 (29) | 15 (25) | 0.69 |
| CCIS | 2 (1–3) | 2 (1–3) | 0.54 |
| ％FVC | 79 (69–93) | 75 (65–88) | 0.20 |
| ％DLco | 59 (45–71) | 62 (43–72) | 0.96 |
| HRCT | | | |
| UIP pattern, N (%) | 50 (71) | 41 (68) | 0.70 |
| Probable UIP pattern, N (%) | 9 (13) | 3 (5) | 0.12 |
| GAP score | 3 (3–4) | 4 (2–4) | 0.11 |
| Anti-fibrotic agent | | | 0.60 |
| From diagnosis to starting AFT, days | 264 (28-618) | 236 (108-753) | 0.43 |
