# Supplementary material for: The clinical impact of comorbidities among patients with idiopathic pulmonary fibrosis undergoing anti-fibrotic treatment: A multicenter retrospective observational study
Source: PLoS One. 2023 Sep 19;18(9):e0291489. doi: 10.1371/journal.pone.0291489 (PMC10508598; doi:10.1371/journal.pone.0291489)
Supplement: Attachment — Submitted filename: Reviewer Comment Re-revision 20230806.docx [file pone.0291489.s007.docx]

**Reviewer #3**

**Manuscript ID**

PONE-D-22-31881R1

**Manuscript Title**

The clinical impact of comorbidities among patients with idiopathic pulmonary fibrosis undergoing anti-fibrotic treatment: A multicenter retrospective observational study

**Article Type**

Research Article

**Journal**

*PLOS ONE*

Thank you for taking the time to review our manuscript and for providing your valuable opinions.

We have reviewed the text carefully and have answered the comments as follows.

**Comment 1**

**Please include sample size (sample size calculation) as this can affect the power of the study.**

**Our reply**

Very important comment, however, no statistical sample size calculations were conducted because of the retrospective nature. So, we inserted the sentences into “Statistical analysis” and “limitation” sections.

**Comment 2**

**You defined disease out come as " IPF-related mortality due to 142 respiratory failure or the first AE within 3 years after initiating AFT", Not clear how you defined AE, in my opinion, you should have kept death and respiratory failure, if need be you could add SAE or AE that may have led to stoppage or change of medication.**

**Our reply**

Thank you for the important comment. In the manuscript, the definition of AE was unclear. So, we re-defined AE as acute respiratory deterioration requiring steroid pulse therapy adding on AFT including clinical worsening of dyspnea, hypoxemia, or the worsening or severe impairment of gas exchange characterized by new bilateral ground-glass opacification/consolidation superimposed on a background pattern consistent with IPF pattern not fully explained by cardiac failure or fluid overload [4]. From the randomized control trial evaluating pirfenidone and nintedanib, the event of AE was very important endpoint [8, 9] and we define as the endpoint as 3-year IPF-related events including IPF-related mortality due to respiratory failure or the first AE within 3 years after initiating AFT. Also, we evaluated the survival curves for 3-year IPF-related mortality according to CCIS in Supplement Figure 2 and demonstrated that comparison of the curves by log-rank test according to CCIS group (A), and according to CCIS group in patients treated with nintedanib (B) and pirfenidone (C) showed no significant difference (P = 0.30, P = 0.21, and P = 0.91, respectively).

**Comment 3**

**Is there a reason you used 'opt out' instead of 'opt-in' in the consent?**

**Our reply**

In this study, there are many death cases, it may be difficult to obtain direct consent from blood relatives, and we would like to analyze all cases that underwent AFT as much as possible. After discussion with the IRB at each institution, opt-in was used.

**Comment 4**

**Remove information in the 'conclusion/discussion ' that you have not provided data in the text.**

**Our reply**

We remove the sentence “(CCIS of GAP stage I, 2 points; II, 2 points; III, 2.5 points (P = 0.32))” and “Furthermore, the different launch dates of pirfenidone (in 2008) and nintedanib (in 2015) in Japan may have contributed to be the limited number of prescriptions, as shown in the supplement figure 2.” in the discussion and moved to results section.

**Comment 5**

**'The long period of AFT is a predictor of outcome'- is this a factor of long AFT or the disease period?**

**Our reply**

Sorry, the sentence was confused. We inserted the sentence, “Especially, in the low CCIS group but not the high CCIS group, the longer duration of AFT had better disease outcome.” In the Abstract section.

**Reviewer #4**

**Manuscript ID**

PONE-D-22-31881R1

**Manuscript Title**

The clinical impact of comorbidities among patients with idiopathic pulmonary fibrosis undergoing anti-fibrotic treatment: A multicenter retrospective observational study

**Article Type**

Research Article

**Journal**

*PLOS ONE*

Thank you for taking the time to review our manuscript and for providing your valuable opinions.

We have reviewed the text carefully and have answered the comments as follows.

**Comment 1**

**In the statistical analysis part, it should be mentioned how checking of the data for normal or non-normal distribution was done. (Ex: Using tests like Shapiro-Wilk or Kolmogorov-Smirnov etc..). This would point to and strengthen the rationale for using mean and SD or median and IQR for continuous variables.**

**Comment 2**

**It is not clear whether the distribution of CCIS scores overall was normally distributed or not. If not, then the criteria for splitting into low and high score groups should be done by their median and not by their mean value. (Lines 172-174).**

**Our reply**

**These comments were very important. We inserted the sentence in the Statistical Analysis section as follows.**

**―**Groups were compared using chi-square test and Wilcoxon rank-sum test (non-parametric test) because a normal distribution cannot be assumed for data obtained with a small number of samples.

―The enrolled patients were divided into the low CCIS group (≤ 2 points) and the high CCIS group (≥ 3 points), because the average and median values of CCIS from our data were 2.2 and 2 points, respectively.

**Comment 3**

**The study has failed to show any difference in outcomes between low and high CCIS groups of IPF patients. However, absence of proof should not be taken as proof of absence. Moreover, even if there had been a difference, it would have been an association and not necessarily a causation. So the conclusion must be carefully written, something like, "In our study, we could not show any relation between CCIS scores and IPF disease outcomes in patients undergoing treatment with anti-fibrotics."**

**Our reply**

Very important comment. We inserted the sentence to the conclusion section as you indicated as follow.

“In the present study, we could not show any relation between CCIS and IPF disease outcomes in patients undergoing AFT, though the longer duration of AFT might be beneficial for IPF outcomes among patients with low CCIS.”

**Comment 4**

**Finally, it was not mentioned whether any patients on nintedanib or pirfenidone faced any complications which prompted a change in dosing or a switch over to the other drug. The pill burden also is usually more for pirfenidone than nintedanib. A lot of patients who suffer from GI complications with pirfenidone or are not comfortable with the pill burden have to be switched over to nintedanib. Such a change however will cause more bias introduced by the treatment giver in the results.**

**Our reply**

Very important comment. We added the information of the number and proportion of AFT dose reduction and discontinuation patients. And in the present study, there was no switch between nintedanib and pirfenidone.

**Reviewer #5**

**Manuscript ID**

PONE-D-22-31881R1

**Manuscript Title**

The clinical impact of comorbidities among patients with idiopathic pulmonary fibrosis undergoing anti-fibrotic treatment: A multicenter retrospective observational study

**Article Type**

Research Article

**Journal**

*PLOS ONE*

Thank you for taking the time to review our manuscript and for providing your valuable opinions.

We have reviewed the text carefully and have answered the comments as follows.

**Comment 1**

**You responded to all question very well. But I am not sure that the question of research is appropriate. Until know the benefit of antifibrotic agent on pulmonary fibrosis is modest. So discussion about effect of antifibrotic agent on comorbidities is not make sense.**

**Our reply**

Thank you for your comment. I agree with your opinion which the benefit of antifibrotic agent on pulmonary fibrosis is modest. We think that the progression of IPF seems to cross-talk with other comorbidities and the longer duration of AFT might be beneficial for IPF outcomes among patients with low CCIS from our data. Therefore, in the present study, we would like to emphasize the importance of evaluating comorbidities in patients with IPF and the optimal use of AFT accordingly may improve the long-term prognosis of IPF.
